# Use of Social Determinants of Health Screening among Primary Health Care Nurses of Developed Countries: An Integrative Review

**DOI:** 10.3390/nursrep13010020

**Published:** 2023-02-07

**Authors:** Deirdre A. McGowan, Carey Mather, Christine Stirling

**Affiliations:** 1 School of Nursing, College of Health and Medicine, University of Tasmania, Glebe, TAS 7000, Australia; 2 Australian Institute of Health Services Management, College of Business and Economics, University of Tasmania, Launceston, TAS 7250, Australia

**Keywords:** health, health screening, integrative, nurses, primary care, social determinants of health, review, systematic

## Abstract

The aims of the study are to evaluate and synthesise research that has investigated social determinants of health screening by primary healthcare nurses; how and when primary health care nurses perform social determinants of health screening; and implications for advancing nursing practice. Systematic searches in electronic databases identified fifteen published studies which met the inclusion criteria. Studies were synthesised using reflexive thematic analysis. This review found little evidence of primary health care nurses using standardised social determinants of health screening tools. Eleven subthemes were identified and collapsed into three main themes: organisation and health system supports are required to enable primary health care nurses; primary health care nurses are often reluctant to perform social determinants of health screening; and the importance of interpersonal relationships for social determinants of health screening. The social determinants of health screening practices of primary health care nurses are poorly defined and understood. Evidence suggests that primary health care nurses are not routinely using standardised screening tools or other objective methods. Recommendations are made for valuing therapeutic relationships, social determinants of health education and the promotion of screening by health systems and professional bodies. Overall, further research examining the best social determinant of health screening method is required.

## 1. Introduction

The social determinants of health (SDH) are the social, political, economic, and ecological factors that contribute to the health and wellbeing of a person, their family, and the community [1]. The evidence for the correlation between SDH and a person’s health and wellbeing is indisputable [2]. When governments and health systems incorporate SDH into policies they reduce social inequalities and disadvantages, create fair living conditions, and support people to live healthy and meaningful lives [1,2,3].

Nursing clinicians comprise over half of the global health workforce [4]. They act across the life cycle and provide care to a broad range of people in diverse settings. Because primary health care is the first and ongoing interaction most people have with health services, primary health care nurses (PHNs) are well placed to identify SDH needs, provide interventions and be powerful influencers in building healthy communities. However, a recent systematic review found nurse clinicians were not performing SDH screening in primary healthcare [5]. Instead, screening was performed by medical providers (40%), research assistants or volunteers (20%), clerical staff (5%) or individuals who were not identified (40%). Primary health care nurses may have performed screening in this latter group; however, there was insufficient reporting, potentially reflecting a lack of consensus around the implementation of SDH screening [5]. Given nurse prominence in the health workforce, it is important to understand whether nursing clinicians ask clients about their social needs and if and how they are using SDH screening tools. This review aims to evaluate and synthesise research that has investigated SDH screening by PHNs, to identify how and when PHNs are performing SDH screening and the implications for advancing nursing practice.

## 2. Background

At the fortieth anniversary of the seminal Declaration of Alma-Ata in 2018 there were renewed calls for high-quality equitable primary healthcare that is comprehensive, integrated, and delivered by skilled, motivated health professionals [6]. Strong primary healthcare systems reduce hospital admissions, reduce inequalities, and improve equitable access to healthcare [7,8]. Yet, a recent Organisation for Economic Co-operation and Development (OECD) report highlighted the need for new models of care to realise the potential of primary healthcare, including broader client participation and financial incentives [9]. Reducing social inequalities and disadvantages to make communities and countries healthier and reduce disease burden on citizens and health infrastructure are key avenues for policy makers and governments to reduce costs and improve outcomes [9].

SDH screening involves asking people about their social needs and concerns, identifying risk and protective factors, and refer to community or specialist services [10]. Clinicians are encouraged to be alert to clinical flags and client cues that indicate the need for SDH screening of clients with specific health conditions, demographic characteristics, or social needs [10,11]. There is limited research from low-income countries, with most research arising from high-income nations [3]. Despite the growing interest and research in SDH screening tools, there remains a lack of consensus about which SDH to screen for, which tool to use, who should conduct screening and how screening should be performed and implemented in clinical practice [5,10,11,12,13]. 

Previous research [3,12,13] identified that clinicians are predominantly screening for one SDH domain and, to a lesser extent, multiple SDH domains (more than one SDH), through a variety of different SDH screening tools [12,13]. One-domain SDH tools exclusively screen for one social factor; whereas, multi-domain SDH tools comprehensively screen for multiple factors [3,13]. A recent systematic review reported that SDH screening tool reliability and validity varied in the literature; 67% reported reliability or validity and only 44% provided evidence through Cronbach alpha scores [13]. Sokol et al.’s [12] systematic review identified inconsistent reporting of paediatric SDH screening tools development.

There is lack of evidence about SDH screening, such as how many SDH to screen for, how frequently to screen, and which tool(s) to use [3,13]. Because SDH needs are often grouped together, O’Brien [13] suggests that multi-domain screening tools are more comprehensive and minimise the need for clinicians to use multiple tools. There is also a lack of consensus on clinician approaches to performing SDH screening, which has been reported as being completed in a variety of ways including pencil and paper, electronic health record, face-to-face interview, telehealth, before or during medical visit, or in the waiting room [5,12].

The literature shows a variety of health care professionals undertake SDH screening, from medical providers, clerical staff, volunteers, nursing clinicians, medical assistants, medical interns, and students [5,14,15]. Although healthcare workers and clinicians support SDH screening and recognise the benefits of holistic care [12,13,16], clinicians are not routinely screening clients [16,17]. Common barriers reported include discomfort and lack of time, knowledge, and confidence [16,17]. PHNs are well placed to address SDH and nurse leaders advocate for greater attention to SDH in baccalaureate programs and nursing practice [18,19]. Yet, it is not clear if PHNs are screening clients for SDH [5].

There is a gap in the research examining what screening PHNs are undertaking, the perspectives of PHNs and the methods they use to identify adverse SDH. The objectives of this study are to systematically identify objective and subjective measures PHNs use to screen for SDH; to identify when PHNs screen for SDH; and to identify the implications for nursing practice. The Population, Concept, Context (PCC) methodology [20] was used to develop the research question and guide the review. Therefore, the research question for this integrative review is how and when PHNs in developed nations perform SDH screening?

## 3. Materials and Methods

Pragmatism underpinned this study and is suited to integrative reviews. Pragmatism focuses on the research question when examining heterogeneous studies, is an iterative process, and enables the researcher to use the most appropriate methods to answer the research question [21,22]. To provide methodological rigour the five stage method outlined by Whittemore and Knafl [23] of problem identification, literature search, data evaluation, data analysis, and presentation was used.

Six electronic databases were searched by the primary reviewer (DM). These were CINAHL, Web of Science, Embase, Medline, PsychINFO, and Scopus from January 2010 to October 2020 using a search protocol in consultation with a research librarian. SDH screening is gaining interest in health research, yet is a relatively novel intervention for healthcare workers [3], thus the search was limited to 10 years from the time of search commencement. The protocol was developed using an integrative review method [23,24] and used key words, controlled language terms and the Boolean operators “or” and “and”. The main search terms were social determinants of health, screening and nurs* (Supplementary Table S1). Grey literature (Google Scholar) was searched following the same protocol. PHNs were defined as registered nurses and nurse practitioners working in primary healthcare. Developed economies were chosen as their health systems are comparative to the Australian context and were identified according to the World Economic Situation and Prospects (WESP) [25] report. The search results were imported into Endnote X9.3.3 for initial title and abstract screening. Inclusion and exclusion criteria were applied to the screening process (Supplementary Table S2). Titles and abstracts were screened for inclusion by the primary reviewer (DM) and scrutinised by two reviewers (CM, CS) and any disagreements were discussed between the reviewers. Once studies were identified for possible inclusion, the full texts of selected studies were reviewed by two reviewers (DM, CS). Any disagreements were discussed between reviewers and a third reviewer (CM) was involved to help reach consensus as required.

**Figure 1:**
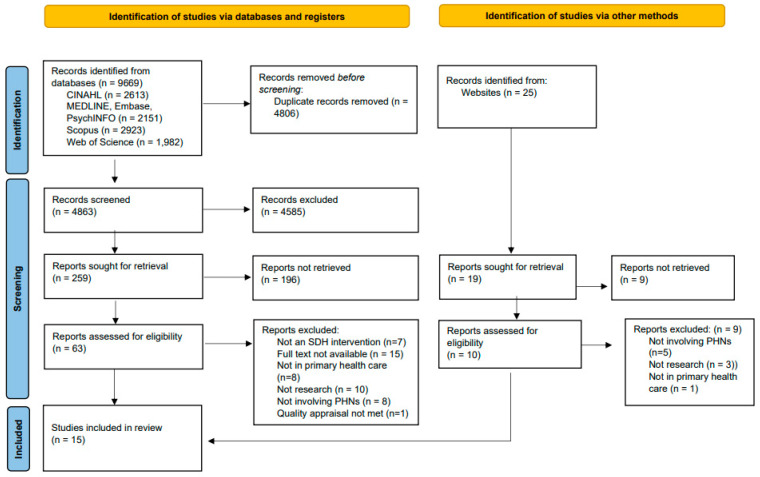
PRISMA flow diagram.

The initial search identified 9694 citations. Following the removal of duplicates, 4806 unique citations were identified. After title and abstract review, seventeen studies were assessed for inclusion at full-text review. Studies were excluded if they did not screen for SDH, were not involving PHNs, were not set in primary healthcare, full not available or if they were theoretical, commentaries, patient case reports or not research. The reference lists of selected studies were hand-searched and one study was identified. Of these studies, sixteen were relevant and underwent quality appraisal. The Preferred Reporting Items for Systematic Reviews and Meta-Analysis (PRISMA) diagram outlines the review process and search outcome [26] (Figure 1).

The Mixed Methods Appraisal Tool (MMAT) [27] was used to appraise the quality of quantitative, qualitative and mixed-methods studies. The Joanna Briggs Institute Checklist for Systematic Reviews [28] was used to appraise systematic reviews (Supplementary Table S3). Two researchers (DM, CS) performed the quality appraisal, any disagreements were discussed between the reviewers and a third reviewer (CM) was available to help reach consensus as required. One study did not meet the quality appraisal criteria of the JBI Checklist for Systematic Reviews [28] and was excluded from analysis. The remaining fifteen studies were of sufficient quality to be included in the review and underwent data extraction.

Data was extracted using the methodology outlined by Whittemore and Knafl [23]. The diverse research was classified into logical and manageable subgroups: quantitative, qualitative, mixed-methods and systematic review studies. Data was extracted into an Excel V16.53 spreadsheet, with each subgroup requiring separate metrics. Data extracted included citation; country; setting; context; research question and aim; study design; target and recruited population; which SDH domain screened for; SDH screening method; screening tool used; when screening was occurring; interventions occurring from screening; study findings; and implications for nursing practice. The included studies are summarised in Table 1.

Reflexive thematic analysis (TA) was chosen to synthesise the data to generate themes from heterogeneous data [29,30]. To provide methodological rigour, a six step structured process outlined by Braun and Clarke [30] was followed: data familiarisation; coding; initial theme generation; developing and reviewing subthemes and main themes; refining, defining and naming subthemes and main themes; and report writing [29,30]. The approach met Braun and Clarke’s [30] criteria for reflexive TA: an inductive versus deductive process was used resulting in codes and themes emerging from the data, rather than applying a lens to interpret the data. The coding and theme generation process was organic, with no utilisation of codebooks or a coding framework. Codes were generated through familiarisation with the data, mind maps, and initial subthemes were created. There was ongoing reflection and time was spent thinking about the data, codes, and themes. The subsequent generation of main themes arose from the subthemes and were identified by the primary reviewer (DM). All reviewers (DM, CM, CS) met on a regular basis to discuss members’ perspectives on subthemes and themes, with the aim of understanding different perspectives and gaining consensus. Interpretation of the findings was checked [31] by two nurse research staff at the School of Nursing, University of Tasmania. Data synthesis resulted in 28 codes that were refined into eleven subthemes and then into three main themes. The main themes are: organisational and health system supports are required to enable PHNs; PHNs are often reluctant to perform SDH screening; and the importance of interpersonal relationships for SDH screening. 

**Table 1:** Summary of included studies.

Author/Year	Location	Research Aim	Design	Sample Size	SDH Identification Method	Key Findings
Amiri and Zhao [32]	Community-based intervention, Uniontown, Alabama United States of America	To describe the nursing observation, assessment, and intervention of an environmental justice community	Mixed methods community-based participatory research (CBPR) model Community mapping Focus group Health survey Kitchen sink water sampling	*n* = 23 residents *n* = 59 drinking water samples	Environmental and demographic community mapping; visiting different places in the community; community consultation and focus groups; health status questionnaire; kitchen sink water sampling	Community members main concern was coal ash and health concerns related to coal ash exposure Community not aware of state resources Drinking water contaminated with lead Nurses collaborating with and advocated for the community
Barboza et al. [33]	Proportionate extended postnatal home visit program, Rinkeby Community Health Centre, Stockholm Sweden	To identify the content of meetings between families and child health nurses during a home visiting program to increase understanding of how the program adds to proportionate universalism	Qualitative content analysis of child health nurses documented home visits	*n* = 98 first time parents	Documentation template with embedded flexibility for parents’ questions, concerns, or issues Emphasis on building trust to encourage families to ask questions about needs or concerns Occurring during the home visit encounter Each visit had a health promotion theme	3 main categories with 12 subcategories: The healthy child: health; care; and development Strengthening the new family: promote mothers health; promote mothers and fathers’ active role; and establish relationship between parents and child Influence and support in the external context: family background and situation; societal structures and resources; medical care; support network; plans and initiatives; and homeland culture and customs
Brooks et al. [34]	One community and mental health NHS Trust Southern England	Identify nurses and allied health professionals practice in identifying and responding to the health literacy needs of patients Clinician’s views about using health literacy screening tools and universal precautions in clinical practice	Qualitative Focus groups Purposive sampling	*n* = 22 (5 nurses, 6 occupational therapists, 4 clinical psychologist, 4 physiotherapist, 1 occupational therapy assistant, 1 exercise rehabilitation instructor)	Tacit cues from clients, such as reading level, body langue, facial expressions, level of engagement in the encounter and clients use of simple language Indirect ways of asking about reading and writing levels Occurring at clinical encounters, such as medication review or routine follow-up phone calls	Clinicians embarrassed Importance of building trusting relationships Participants stated they had knowledge of health literacy strategies, yet rarely utilised these Health literacy assessment was viewed as redundant if care was not tailored Tailoring information was regarded as too costly and time consuming Intimidated by people with high health literacy Some clinicians believed health literacy screening was unnecessary in their area or caused unnecessary stress Concerned about offending patients Not using health literacy screening tools “I wouldn’t want to [use screening tool]” Barriers identified: Administrative and time constraints
Browne-Yung et al. [35]	One sleep disorder clinic and one anxiety clinic, Adelaide, South Australia Australia	To understand clinicians’ and patients’ views on SDH tools and develop a brief social health history (SHS) screening tool	Qualitative Framework analysis over two stages: Stage 1—Development of a SHS screening tool Stage 2—Refining and testing the SHS tool	Stage 1: Consumers and consumer advocate focus group *n* = 6 Clinician interviews *n* = 16 Stage 2: Consumer and consumer advocate focus group *n* = 5 Clinicians *n* = 12 Patients *n* = 50	Self-completed by patient prior to first appointment, mailed out and completed at home	Stage 1: Consumer advocates positive about SHS tool as a conversation starter Tool should avoid duplication of information already collected Occupation and visual cues used by clinicians prior to SHS tool Clinicians too busy to complete SHS tool face-to-face Clinicians value collection of SHS information Stage 2: No adverse SDH identified secondary to small sample size SHS tool took 6-min to complete Patients and clinicians had positive attitudes towards screening Some participants felt they might not be as comfortable if they had adverse SDH Clinicians had mixed feelings of usefulness of information ‘Did not influence care with this patient…’ and ‘…Helped plan session especially around mood’ and ‘We also obtain similar information—may be useful for places with no means to obtain this info’
Dodge et al. [36]	Maternal, Infant and Early Childhood Home Visiting Program, North Carolina United States of America	Evaluation of a universal, postnatal nurse home-visiting intervention for penetration, fidelity, emergency healthcare episodes, and positive parenting by 6 months of age Hypotheses: Reach most birthing families with high fidelity and cost effectiveness Improve family connections to community resources Improve parenting and family function Improve infant outcomes	Randomised control trial	*n* = 4777 Intervention group *n* = 2327 (even birth date) Control group *n* = 2450 (odd birth date) Random subsample blinded interviews *n* = 531	4–7 scripted contacts, risk assessment scoring tool and rapid triage Recruitment screen at birthing hospital and ongoing screening at home visit Occurring face-to-face and telephone contact	Program completion rate 85.8% and fidelity of 84% 99% of intervention participants would recommend intervention Intervention participants had 59% fewer infant emergency medical care episodes than control participants Emergency care episodes differed from first month after intervention and increased with time demonstrating an immediate and deferred effect Intervention group had 0.86 more community resources than control Intervention mothers reported more positive parenting behaviours Blinded observers rated quality of home significantly higher for the intervention group Intervention mother less likely to report clinical anxiety Health cost - benefit ratio of 1:3.02
Galletly et al. [37]	Mental health community and outpatient setting, Adelaide Australia	To investigate health literacy in people with mental health and evaluate the relationship between medication adherence and health literacy. Hypothesis – Those with schizophrenia or major depression have impaired health literacy levels compared to the general population	Quantitative descriptive study Assessment interview and self-assessment	*n* = 60 (schizophrenia *n* = 30; major depression *n* = 30)	Self-assessment using Test of Functional Health Literacy in Adults (TOFHLA) at commencement of research	No correlation between health literacy and medication adherence (r = −0.05, *n* = 60, *p* = 0.697. 93% of major depression and 97% of schizophrenia participants scored ‘adequate’ for Health literacy
Godecker et al. [38]	Primary health care clinic, Minneapolis-Saint Paul United States of America	To identify if there are any differences in identifying Prenatal Risk Overview (PRO) risk factors between registered nurses and community health workers	Non-randomised control trial	*n* = 733 prenatal women	Prenatal Risk Overview (PRO) structured screening interview performed during the prenatal intake appointment	Registered nurses and community health workers had similar risk identification results for 6 of 12 domains Community health workers identified more High-Moderate Risk and High Risk for telephone access (*p ≤* 0.001), transport access (*p ≤* 0.05), food insecurity (*p ≤* 0.01), housing instability (*p ≤* 0.001), social support (*p ≤* 0.05) and depression (*p ≤* 0.01). Registered nurses identified more High Risk for alcohol use (*p ≤* 0.05) Community health workers elicited more subjective responses Registered nurses applied more clinical judgement
Gruβ et al. [39]	Community health centres from OCHIN network, across five states (Oregon, California, Minnesota, Indiana, North Carolina) United States of America	Understand factors that hinder or facilitate implementation of electronic health record (EHR)-based SDH screening into workflows at Community Health Centres across the USA by interviewing CHC staff.	Formative qualitative sub study Year 1 of 5 year ASCEND study (Approaches to Community health centre implementation of social determinants of health Data collection and action)	*n* = 52 clinicians and health workers (12 administrators, 2 informatics staff, 12 clinical staff, 7 behavioural health staff, and 10 community health workers)	OCHIN EHR-based screening questionnaire	Three themes emerged that enable organisations and clinicians: External incentives and motivators Internal SDH screening advocates with allocated time and resources for workflow solutions, promote uptake of screening amongst staff, provided feedback to staff and developed materials Maintaining flexible attitudes about workflows to optimize clinic needs, interests, and resources
Hornor et al. [40]	Mixed settings: 66% in primary care, 28% hospital-based or community-based specialty practice, 7% in acute care United States of America	To describe paediatric nurse practitioner practice behaviours related to screening and providing anticipatory guidance for child maltreatment and its psychosocial risk factors	Descriptive quantitative Survey	*n* = 243 paediatric nurse practitioners	Self-reported clinical practice behaviours at every visit, well-child visit, initial visit, or symptom specific visit	Paediatric nurse practitioners are not routinely screening children and parents for child maltreatment and its risk factors 51% never/rarely ask parents about domestic violence 30% never/rarely ask parents and 44% never/rarely ask children about discipline practices at home 50% perform ano-genital exam at well-child visit Multiple barriers identified: time constraints (67%), lack of training (48%), feeling uncomfortable (32%), lack of validated evidence-based screening tools (29%) and not in their scope of practice (4%)
McCune et al. [41]	7 primary care practices, Midwest (3 inner city, 2 university towns, 2 clinics in rural and urban areas) United States of America	To examine and explore provider-staff awareness and perceptions of patient health literacy status within the primary care setting To test implementation of the Newest Vial Sign (NVS) screening tool to measure health literacy; gain sample percentages of health literacy; examine administration; and examine patient perspectives	Cross-sectional exploratory study Patient survey Clinicians focus group Clinician predictions of literacy level	*n* = 282 patient population *n* = 47 clinician and clinic staff	Newest Vital Sign (NVS) Completed with routine vital signs at 3 sites and in the waiting or examination room immediately after their visit at 4 sites	2 of 7 (28.6%) sites predictions of literacy level correlated with assessed literacy levels Patient and clinician positive attitudes to health literacy screening Clinicians expressed reluctance to screening due to adding an extra task and time constraints NVS took 3–5 min to complete
Monsen et al. [42]	Mixed settings: community, ambulatory and acute care United States of America	To examine the documentation of social and behavioural determinants of health (SBDH) in electronic health records (EHR) with and without standardised nursing terminologies	Comparative study design; convenience sampling In-depth interview	*n* = 9 health- and information-system leaders and experts	Terminology documented in EHR	Identified 107 SBDH phrases, grouped into 24 topics Phrases documented in EHRs using free text, structured text, or standardised terminologies Standardised terminology was documented most often by nurses or other clinical staff compared with non-clinical staff
Purkey et al. [43]	Family medicine and paediatric care, rural and urban regions South-eastern region of Ontario Canada	Evaluate the implementation of a clinical poverty screening instrument in a diverse range of family medicine and peadiatric clinical settings. To examine the uptake of screening for poverty, evaluate acceptability by patients and explore health care practitioners’ experiences of universal implementation of the clinical poverty instrument	Exploratory mixed methods study Clinician self-reported number of patients screened Clinician semi-structured focus group Patient questionnaire	*n* = 22 Clinicians *n* = 18 clinician focus group *n* = 150 patients	Clinical single item poverty screening tool “Do you ever have difficulty making ends meet at the end of the month?” During consultation	Clinician self-reported screening rate 9% Patients had positive attitudes to poverty screening Clinician surprised by screening results and uncovering hidden poverty Clinician barriers identified: changing habits, unsure how to incorporate into their practice, discomfort, lack of expertise Systemic and organisational barriers identified: appointment length and structure, electronic health record could make it both easier or harder and the number of other screening tasks expected to perform, inadequate interventions and resources
Shreffler-Grant et al. [44]	Eight rural communities from two north-western states United States of America	Evaluate the impact of a community-based skill building intervention on complementary and alternative therapies health literacy and general health literacy Hypothesis: Complementary and alternative therapy health literacy scores and general health literacy scores immediately after the intervention will be higher than scores at commencement of intervention and 5-months after the intervention	Pre-test post-test quasi-experimental study design; educational skill building Questionnaire, general health literacy measure, complementary and alternative therapies health literacy measure, demographic questions	*n* = 127 older adults	Newest Vital Sign (NVS) Single item general health literacy measure “How confident are you filling out medical forms by yourself?” Montana State University Conceptual Model of Complementary and Alternative Therapies Health Literacy (MSU CAM) Screening at end of first session, at end of last session, and 5 months after intervention	Complementary and alternative therapies health literacy score was higher immediately after the intervention (mean = 70.67) compared to start of intervention (mean = 68.48) and decreased 5 months after the intervention (mean = 69.9) NVS score increased overall at each stage Single Item measure was higher immediately after the intervention (mean = 3.95) compared with start of the intervention (mean = 3.73) and decreased 5 months after the intervention (mean = 3.90) Health literacy wanes over time Two general health literacy screening tools gave different results when measuring health literacy 5 months after the intervention
Sisler et al. [45]	Simulation experience in an urban health-career education program with graduate nurse practitioner trainees and community adolescents as simulated patients United States of America	Explore how adolescent patient actors in a simulated patient-provider interaction can improve nurse practitioner trainees’ ability to assess and address social needs	Qualitative preliminary research Patient-actor feedback Nurse practitioner group debrief Nurse practitioner self-reflections	*n* = 36 nurse practitioner trainees *n* = 23 adolescent patient actors	CRAFFT Screening Test for substance-related risks and problems in adolescents (Car, Relax, Alone, Forget, Friends, Trouble) HEADSS Psychosocial Interview for Adolescents (Home and environment, Education and employment, Activities, Drugs/Depression, Sexuality, Safety) SSHADESS: A Strength-Based Psychosocial Assessment (Strengths, School, Home, Activities, Drugs/diet, Emotions, Sexuality, Safety).	Low-risk interactions improved confidence in asking questions sensitively and identify SDH needs Five major themes emerged: “If we don’t ask, they often will not share” Considering the root of the problem Balancing patient and provider priorities Developing interpersonal skills Being mindful
Tallon et al. [46]	Various paediatric nursing settings (palliative, community, primary health, oncology, prenatal, and child and family)	Seriously or chronically ill children and families psychological and social circumstances is not well understood in paediatric nursing care	Systematic integrative review	*n* = 13 research articles reviewed		Tension between assessing social needs and medical care 80% of nurses think patients rate physical health over psychosocial health Patients experience stigma when disclosing social needs Multiple clinician barriers to SDH screening: time, available resources, lack of skills, lack of validated tools, lack of confidence, relying on clinicians’ judgement without assistance or infrastructure Not all nurses believe SDH screening is their responsibility, 35% believe nurses are responsible, 33% think social workers are responsible, and 32% think other disciplines are responsible (physician, pastoral care, behavioural healthcare) Indirect strategies such as ‘chatting’ Avoidance and blocking strategies when patients were distressed

## 4. Results

### 4.1. Included Studies 

Fifteen studies published between 2010–2020 which met the inclusion criteria were identified. Studies selected for review were quantitative (*n* = 6), qualitative (*n* = 5), mixed methods (*n* = 3), and systematic review (*n* = 1). The quantitative studies were quantitative descriptive [37,40], non-randomised control trial [38], comparative [42], pre-test post-test quasi-experimental [44] and randomised control trial [36]. The qualitative studies were content analysis [33], focus group [34], framework analysis [35], formative qualitative sub study [39] and qualitative preliminary research [45]. The mixed-method studies were community-based participatory research [32], cross-sectional exploratory [41], and exploratory mixed method [43]. There was one systematic integrative review [46] (Table 1). Studies were from the United States of America (*n* = 9), Australia (*n* = 3), Sweden (*n* = 1), the United Kingdom (*n* = 1) and Canada (*n* = 1). Sample size ranged from 9 participants to 4777 participants (quantitative 9–4777, qualitative 22–98, mixed-methods 28–334 and systematic review 13 studies) (Table 1). 

Six studies identified PHN use of SDH screening tools in primary care practice [41], paediatric and family health [43], community mental health [37], community-based participation [44], sleep disorder and anxiety clinics [35] and graduate nurse practitioner training [45]. Three studies screened for health literacy via the Test of Functional Health Literacy [37,47], the Newest Vital Sign [41,44,48], the single item general health literacy measure question (“How confident are you at filling out medical forms by yourself?”) [44,49], and the Montana State University (MSU) Conceptual Model of Complementary and Alternative Therapies (CAM) Health Literacy (MSU CAM) [44,50]. The remaining studies utilised the social health screening tool [35], clinical poverty screening tool single item question (“Do you ever have difficulty making ends meet at the end of the month?”) [43,51], and adolescent health tools (CRAFFT screening test for substance-related risks and problems in adolescents, HEADSS Psychological Interview for Adolescents, and SSHADESS a Strengths Based Psychosocial Assessment) [45,52,53,54]. Selected studies varied in the delivery of SDH screening with multiple methods reported. The most common method was face-to-face screening during a consultation or interview, occurring in three studies [37,43,45]. One study mailed the screening tool to clients for completion at home prior to their first visit [35] and one study used a mixture of locations, such as the waiting room, examination room and during vital signs [41]. One study used a mixture of on-site screening pre- and post-test and mail out for five-months post-test screening [44] (Table 1).

Five studies used other methods to identify multi-domain SDH needs, such as scripted interviews, risk assessment and triage [36], documentation templates with embedded flexibility [33], Prenatal Risk Overview (PRO) screening interview [38], community-based participatory approach with the aid of community mapping and environmental justice assessment [32], and iterative approaches to find the best fit [39]. These other methods were used in community health centres [39], community-based participation [32], postnatal home visiting programs [33,36], and prenatal primary healthcare [38]. One study found clinicians used indirect methods such as tacit cues and body language to assess health literacy [34], another study examined self-reported paediatric nurse practitioner clinical behaviours [40] and another study identified electronic health record standardised nursing terminology use [42] (Table 1).

### 4.2. Themes

The research aims were to evaluate and synthesise research that investigated SDH screening by PHNs, how and when PHNs perform SDH screening, and its implications for advancing nursing practice. The results found little evidence of PHNs using SDH screening tools, with only six reviewed studies describing tool utilisation across the contexts of community mental health, family and paediatric community care, primary care practice, nurse practitioner training, community-based skills intervention and sleep disorder and anxiety. PHNs are predominantly using SDH screening tools specific to their organisation and client needs, with no standardisation of practice. Eleven subthemes were identified from the reviewed studies and collapsed into three main themes: organisational and health system supports are required to enable PHNs; PHNs are often reluctant to perform SDH screening; and relationships are an important aspect of screening. Table 2 outlines the codes, subthemes and themes identified from the reviewed studies. The subthemes have been italicised in the main themes.

#### 4.2.1. Organisational and Health System Supports Are Required to Enable PHNs

PHNs’ diverse experience enables and creates barriers to SDH screening at the individual, organizational and health system level, which highlights the need for organizational and health system support to enable PHNs to perform SDH screening [34,35,36,40,41,43,45,46].

One reviewed study reported on factors that *enable* PHNs to incorporate SDH screening into their practice, such as incentives from funding bodies or professional organisations, protected time and workflows to promote SDH screening and flexibility when implementing SDH screening workflows [39]. Monsen et al. [42] compared standardised nursing terminology use in diverse electronic health record systems (primary care clinics, hospitals, and public health) and found that nurse clinicians are more likely to use standadised terminologies when documenting SDH than non-clinical staff. Monsen et al. [42] suggest that SDH standardised nursing terminology can assist PHNs to document their findings, communicate with other health professionals and understand the SDH needs of people in their care.

The most reported *barrier* was lack of time [36,40,41,43,46], followed equally in number by administration barriers such as duplication of information and forms [34,35], lack of clinician training, skill level or validated tools [40,46] and limited availability of community resources to refer patients onwards [43,46]. Gruβ et al. [39] identified three ways to *enable* PHNs to incorporate SDH screening into their practice: (1) incentives from health systems, such as grant requirements, funding, and promotion from peak professional bodies to encourage organisations to provide environments for clinicians to adopt SDH screening into practice; (2) organisations utilising an internal SDH screening advocate who has dedicated time and resources to promote and develop resources and coordinate workflows; and (3) clinicians and organisations having flexible attitudes to their SDH screening approaches. Flexibility enabled clinicians to test different types of screening methods and workflows to find the best fit for the organisation’s interests, resources and client population needs [39].

Using the appropriate screening tool for the population was identified as important by two reviewed studies [37,44] as *screening tools gave inconsistent results*. Galletly et al. [37] found the Test of Functional Health Literacy in Adults (TOFHLA) [47] demonstrated a ceiling effect in populations with adequate to high health literacy, such as Australia. In their study, Shreffler-Grant et al. [44] found the Newest Vital Sign (NVS) [48] and single item general health literacy measure (“How confident are you filling out medical forms by yourself?”) [49] gave different results when measuring health literacy five months after an intervention. Both studies [37,44] suggest the need for further research on the validity of health literacy screening tools in diverse populations and over time.

Adequate workflows and support impact the *feasibility of screening* and success of SDH screening interventions [36,40,43]. Dodge et al. [36] demonstrated universal coverage in a randomized control trial with a postnatal nurse home-visiting program that was short, community owned, economical and had health system support. There was a high completion rate, high fidelity, and a health cost–benefit ratio of 1–3.02 [36]. SDH identification occurred via scripted interviews, risk assessment scoring and rapid triaging face-to-face and telehealth, demonstrating how interventions that are well-designed for the circumstances, support PHNs and align with community resources are effective [36]. Conversely, Purkey et al. [43] implemented a universal clinical poverty tool [51] in the ‘real world’ where minimal organisational and health system support was provided. Family physicians, nurse practitioners and paediatricians attended a three-hour “Treating Poverty” workshop, where they were given instructions on how to use the screening tool and advised to find the best way to implement screening into their clinical practice [43]. In this minimal support environment, universal screening was not achieved, with clinicians self-reporting screening at 9% [43]. Similarly, Hornor et al. [40] found that, despite recommendations for child maltreatment screening, paediatric nurse practitioners are not routinely performing screening in their self-reported practice.

#### 4.2.2. PHNs Are Often Reluctant to Perform SDH Screening

The attitudes and perceptions PHNs have towards SDH screening can result in a reluctance to incorporate SDH screening into their clinical practice. *Clinician attitudes* toward years of clinical experience were viewed as adding to a clinician’s ability to identify adverse SDH [34,41]. However, PHNs and allied health professionals felt that reliance on clinical identification did not always work [34]. Additionally, only 28.6% of PHNs, physicians and medical assistants correctly estimated health literacy when assessed by the Newest Vital Sign [41,48]. Purkey et al. [43] found that PHNs and physicians were surprised to uncover hidden poverty, especially in people with whom they had had therapeutic relationships with for years.

Three reviewed studies [34,43,46] identified that PHNs believe that patients prioritise physical health over psychosocial health [46], do not consider SDH screening relevant for all client groups, believe screening is redundant unless a suitable intervention is provided [34], and question if they would use screening tools for all clients [43]. Additionally, not all PHNs believe SDH screening to be within their scope of practice, with 65% of nursing clinicians and 4% of paediatric nurse practitioners believing it is the role of other health professionals [40,46].

PHNs reported experiencing *discomfort*, embarrassment, and feared offending people when asking sensitive questions, leading to curbing or avoiding sensitive topics [34,45,46]; however, training and education can overcome this situation. Sisler et al. [45] demonstrated the importance of providing safe places for nurse practitioner trainees to practice asking sensitive questions. Brooks et al. [34] found clinicians were intimidated by people with adequate or high health literacy, defining this group as challenging, and as having higher expectations of the information clinicians should provide. In this study, clinicians were concerned about offending or patronising people who have high health literacy and were concerned people would question their professional status if they used simple, accessible language and information [34].

Five reviewed studies [32,35,36,41,43] identified positive *client attitudes* to being asked about SDH needs. Clients felt comfortable, would recommend it to others, and believed screening should occur in clinical encounters [35,36,41]. Although, there is a portion of people who feel discomfort with screening secondary to finding tools difficult, stigma or fear of clinician reactions [35,41,46]. Godecker et al. [38] suggest *client attitudes* and perceptions of PHNs may result in non-disclosure of adverse SDH, perceiving PHNs to be less likely to experience hardships. Godecker et al. [38] compared PHN and community health worker SDH screening interviews and found that community health workers identified different SDH risks. Community health workers identified more subjective responses, such as concerns or worries [38].

#### 4.2.3. The Importance of Interpersonal Relationships for SDH Screening

The studies for review identified the importance of building trusting relationships for successful SDH screening [32,33,45]. For trusting relationships to happen, PHNs need to *move beyond the bio-medical model* to create authentic encounters and *therapeutic relationships.* PHNs also use *interpersonal skills* when screening. Two reviewed studies [45,46] found that PHNs use chatting, verbal and non-verbal cues, client use of simple language and client (dis)interest in the nurse–client encounter. Sisler et al. [45] found graduate nurse practitioner trainee behaviours, such as body language and focusing on writing or typing, can intimidate patients and hinder trusting relationships.

Sisler et al. [45] found that graduate nurse practitioner trainees can build *therapeutic relationships* by developing interpersonal skills, balancing patient–provider priorities, being mindful during encounters and consider the SDH to be the cause of a problem. In this study, the trainees were provided with learning and simulation opportunities to become comfortable with asking sensitive questions, allowing silence within interactions, providing opportunities for clients to express their concerns and engaging in active listening [45].

Community-based participatory approaches were utilised to build relationships with community members in two studies [32,44]. Amiri and Zhao [32] worked with community members to advocate for community environmental pollutants with legislators, senators, and government departments. In this study, community members were provided with workshops about communicating with legislators, key stakeholders and reducing lead level exposure in drinking water [32]. Another study [44] built community skills through a community-based health literacy program.

One reviewed study [33] described the content of PHNs’ home-visiting encounters and identified an emphasis on building trusting relationships through documentation templates that had embedded flexibility to allow clients to express concerns and ask questions.

## 5. Discussion

The aims of this review were to evaluate and synthesise research that investigated SDH screening by PHNs, how and when PHNs perform SDH screening, and its implications for nursing practice. This integrative review provided a range of findings from the reviewed studies, resulting in the generation of three main themes. The implications for PHNs drawn from these themes can be used to guide further research and advance nursing practice.

This review has found little evidence (only six studies) of PHNs using standardised SDH screening tools. A further six studies described PHNs using other objective methods to collect SDH information such as interviews, questionnaires, and templates. There were varied approaches to screening with the delivery ranging from face-to-face, telehealth, during consultation, in waiting rooms, or self-completed at home by the client. The lack of implementation consensus is echoed in the literature [3,10,12] and questions remain about whether techniques such as SDH screening tools, other objective measures, and developing PHNs’ therapeutic relationship skills will increase SDH screening by nurse clinicians [55,56].

Subjective measures, such as interpersonal skills and tacit cues are also used by PHNs; however, this review highlighted a lack of clarity about the appropriate mix and weighting of subjective and objective methods. The use of objective standardised tools are familiar to clients and clinicians, but at times clients can feel uncomfortable or stigmatised by screening [45,57]. Additionally, although standardised SDH terminology assists communication between health professionals [42], it can impede understanding, hinder communication, and remove the person from the experience [58].

In the absence of SDH screening tools, PHNs make judgements, assumptions and, at times, may incorrectly estimate client needs [34,41]. The incorrect estimation of client needs is consistent with research about hospital and acute-based nurse clinician estimation of health literacy [57]. In a systematic review of healthcare providers and clients perceptions of health literacy, nurse clinicians inaccurately estimated the health literacy needs of clients and utilised subjective measurements, such as gut feelings [57]. Clinicians must be cognisant of the fallibility of assumptions or judgement, especially when the factors that contribute to SDH, such as race, ethnicity, gender, disability, and socioeconomic status, are also factors that contribute to implicit bias [59]. These findings suggest that subjective measures should be used carefully and in tandem with objective screening.

The importance of relationships to effectively identify SDH needs was identified and should not be confused with subjective assessment [32,33,45]. Therapeutic relationship skills need to be learned and developed, yet are often unrecognised or viewed as an implicit ability [60]. The components within therapeutic relationships of interpersonal skills, listening, mindfulness, awareness of oneself and empathy promote connections between clinicians and clients and create better health outcomes [61]. Sisler et al. [45] identified the importance of safe environments for nurse practitioner trainees to practice SDH screening, build confidence and develop the skills required for effective SDH screening and therapeutic relationships. Moreover, further research about how to teach PHNs therapeutic relationship skills is required [60]. Support provided to build therapeutic relationships with clients, such as time, resources and valuing long-term relationships is important [39,57]. However, moving towards relationship building and maintenance may require a radical leap for primary healthcare systems that emphasise disease and treatment, fee-for-service and short-term gains [3,62].

Clients mostly support SDH screening [57,63]; however, PHNs are often reluctant to screen for SDH due to clinician, organisational and system barriers [39] such as discomfort, lack of familiarity and confidence with screening, and unsupportive working environments [3]. In a study of hospital-based nurses’ perceived SDH self-efficacy, Phillips et al. [64] found nurses were more confident asking clients about SDH that were related to routine nursing assessments, such as access to healthcare. Additionally, nurses reported a desire for more education and interdisciplinary collaboration to increase knowledge and confidence [64]. While training and education opportunities have the potential to improve confidence in screening for SDH, evidence of actual behaviour change is needed to ensure that programs effectively reduce health disparities [55,56].

To provide comprehensive, person-centered care PHNs require a comprehensive understanding of the importance of SDH on a person’s health and wellbeing. In a systematic review of healthcare clinicians’ attitudes to health literacy, Rajah et al. [57] found clinicians had minimal formal health literacy education, limited understanding of the effects of low health literacy on health and wellbeing, and did not consider health literacy interventions to be a high priority. These findings highlight the importance of improving education about SDH for nurses and PHNs in baccalaureate and continuous professional development programs [15,18,57,64,65].

Dodge et al. [36] achieved universal coverage of a postnatal nurse home-visiting program through utilization of scripted interventions, risk assessment scoring and rapid triage via face-to-face contact and telehealth. Their findings suggest that a SDH screening that is designed appropriately for the purpose, organisation, and community, and which supports PHNs, can be effective [36]. Amiri and Zhao [32] demonstrated SDH advocacy through a community-based participation approach to identify community environmental contaminants, educate community members and advocate with governments and legislators. This suggests that environments that can enable PHNs to implement SDH screening into clinical practice such as having incentives from funding bodies or effective workflows, emphasis on therapeutic relationships and flexible screening practices are more important than the method used [32,39,45]. However, further research is required due to the small sample of studies. PHNs have the potential to become SDH screening advocates and powerful influencers on SDH for clients and their communities [18].

### Limitations and Strengths

This study did not review studies prior to 2010, in languages other than English, where the full text was not available, or studies from undeveloped or in-transition nations; consequently, some studies may have been missed. Studies which were of sufficient quality to be included in the study predominantly had sample sizes of less than 100 and moderate quality of evidence with only one randomized control trial available that met the selection criteria. Five studies included sample populations of multidisciplinary teams where PHNs were members; thus, findings and implications may not be representative of all PHNs. This study did not evaluate and synthesise findings from studies examining nurse clinicians in settings outside of primary healthcare or other health professionals, so potentially it is missing implications for nursing practice. Primary healthcare covers diverse settings, the low number of studies reviewed may not be representative of all primary healthcare settings or PHNs.

There are many strengths to this study. Integrative reviews are suited to nursing practice, enable heterogeneous studies to be reviewed and provide depth and breadth to findings and implications for nursing practice [66]. To promote methodological rigour and transparency the recognised method outlined by Whittemore and Knafl [23] was used. The utilisation of MMAT [27] and the Joanna Briggs Institute Checklist for Systematic Reviews [28] enabled diverse research methodologies to be appraised for quality. Reflexive TA criteria was met through data emersion and familiarisation, mind maps, generation of codes and themes, reflection and consensus [30].

## 6. Conclusions

The SDH screening practices of PHNs are poorly defined and understood. Evidence suggests that PHNs do not routinely use standardised SDH screening tools or other objective methods such as interviews and questionnaires. Results suggest that subjective measures should be used carefully and in tandem with objective measures; however, further research examining the best SDH screening method for PHN use is required. To enable PHNs to ask clients about their SDH needs valuing therapeutic relationships and investment in skill development, such as training, time and resources is required. Nursing education and training can improve knowledge and confidence in performing SDH screening; however, it must be matched with evidence of actual behaviour change. Health systems and professional bodies can promote SDH screening through incentives or funding requirements. There is great potential for PHNs to become SDH screening advocates and expand their practice to bridge health disparities and build healthier communities.

## Figures and Tables

**Table 2:** Themes generated from findings.

Code	Subtheme	Theme
Enabler	Enablers	Organisational and health system supports are required to enable PHNs
Training	Barriers	
SDH tool		
Administration		
Time		
Resources		
Not screening	Screening feasibility	
Universal screening		
Different results	Tools gave inconsistent results	
Relevance	Clinician attitudes	PHNs are often reluctant to perform SDH screening
Education		
Reluctance		
Assumptions		
Practice		
Insult	Clinician discomfort	
Intimidated		
Discomfort		
Embarrassed		
Patient attitude	Patient attitudes	
Patient discomfort		
Terminology	Interpersonal skills	Importance of interpersonal relationships for SDH screening
Indirect		
Interpersonal skills		
Trust	Therapeutic relationships	
Balancing priorities		
Advocacy		
Root of the problem	Move beyond bio-medical model	

## Data Availability

Data available in article Supplementary Materials.

## Supplementary Materials

The following supporting information can be downloaded at: https://www.mdpi.com/article/10.3390/nursrep13010020/s1, Table S1: Integrative review search strategy; Table S2: Screening criteria; Table S3: Quality appraisal.
