# Supplementary material for: Use of Social Determinants of Health Screening among Primary Health Care Nurses of Developed Countries: An Integrative Review
Source: Nurs Rep. 2023 Feb 7;13(1):194–213. doi: 10.3390/nursrep13010020 (PMC9944459; doi:10.3390/nursrep13010020)
Supplement: Supplementary file 1 [file nursrep-13-00020-s001.zip › SuppInfo Table S1.pdf]

Supplementary Table S1: Integrative review search strategy
**CINAHL database:**<br>social determinants of health OR social determinants OR housing insecurity OR poverty OR social isolation OR health inequalities OR psychosocial OR socioeconomic status OR health literacy OR population health OR MeSH: Social Determinants of Health<br>AND<br>Screening OR screening tools OR assessment OR assessment tools OR assessing OR<br>intervention OR consultation OR care planning OR conversation OR community health assessment OR MeSH: Health Screening<br>AND<br>Nursing OR nurse OR nurses OR nurse clinician OR nurse specialist OR MeSH Clinical<br>Nurse Specialists
**Embase and PsychINFO (via Ovid) databases:**<br>social determinants of health OR social determinants OR housing insecurity OR poverty OR<br>social isolation OR health inequalities OR psychosocial OR<br>socioeconomic status OR health literacy OR population health OR Subject heading: Health<br>disparities; socioeconomic status; sociocultural factors; and psychosocial factors<br>AND<br>Screening OR screening tools OR assessment OR assessment tools OR assessing OR<br>intervention OR consultation OR care planning OR conversation<br>OR community health assessment OR Subject heading: Screening<br>AND

Nursing OR nurse OR nurses OR nurse clinician OR nurse specialist OR Subject heading:<br>Nurses
**Medline (via Ovid) databases:**<br>social determinants of health OR social determinants OR housing insecurity OR poverty OR social isolation OR health inequalities OR psychosocial OR socioeconomic status OR health literacy OR population health OR MeSH: Social determinants of health; Health status; Health status disparities; Health status indicators<br>AND<br>Screening OR screening tools OR assessment OR assessment tools OR assessing OR intervention OR consultation OR care planning OR conversation OR community health assessment OR MeSH: Health surveys<br>AND<br>Nursing OR nurse OR nurses OR nurse clinician OR nurse specialist OR MeSH: Nurses
**Web of Science and Scopus databases:**<br>social determinants of health OR social determinants OR housing insecurity OR poverty OR social isolation OR health inequalities OR psychosocial OR socioeconomic status OR health literacy OR population health<br>AND<br>Screening OR screening tools OR assessment OR assessment tools OR assessing OR intervention OR consultation OR care planning OR conversation OR community health assessment<br>AND<br>Nursing OR nurse OR nurses OR nurse clinician OR nurse specialist
