# Supplementary material for: Use of Social Determinants of Health Screening among Primary Health Care Nurses of Developed Countries: An Integrative Review
Source: Nurs Rep. 2023 Feb 7;13(1):194–213. doi: 10.3390/nursrep13010020 (PMC9944459; doi:10.3390/nursrep13010020)
Supplement: Supplementary file 1 [file nursrep-13-00020-s001.zip › SuppInfo Table S2.pdf]

Supplementary Table S2: Screening Criteria

Inclusion	Exclusion
Qualitative, quantitative, and mixed methods research<br>Published in the English language<br>Publish date between 2010 – 2020<br>Full text available<br>Research conducted from a developed nation<br>Research investigating SDH screening by PHNs and multidisciplinary teams where PHNs are members<br>Research in primary health care	Research which is unpublished<br>Publish date before 2010<br>Published in languages other than English<br>Full text not available<br>Research where screening is performed by other health professionals (not nurses)<br>Research in tertiary or secondary health care<br>Theory, commentary, or patient case reports<br>Research where the predominant focus is on behavioural modification, lifestyle factors, or psychosocial counselling interventions
