# Supplementary material for: Use of Social Determinants of Health Screening among Primary Health Care Nurses of Developed Countries: An Integrative Review
Source: Nurs Rep. 2023 Feb 7;13(1):194–213. doi: 10.3390/nursrep13010020 (PMC9944459; doi:10.3390/nursrep13010020)
Supplement: Supplementary file 1 [file nursrep-13-00020-s001.zip › SuppInfo Table S3.pdf]

Supplementary Table S3: Quality Appraisal

CITATION: AMIRI AND ZHAO (2019) 2
CITATION: BARBOZA ET AL. (2018) 3
CITATION: BROOKS ET AL. (2020) 4
CITATION: BROWNE-YUNG ET AL. (2019) 5
CITATION: DODGE ET AL. (2014) 6
CITATION: GALLETLY ET AL. (2012) 7
CITATION: GODECKER ET AL. (2013) 8
CITATION: GRUSS ET AL. (2020) 9
CITATION: HORNOR ET AL. (2017) 10
CITATION: MCCUNE ET AL. (2016) 11
CITATION: MONSEN ET AL. (2019) 12
CITATION: PURKEY ET AL. (2019) 13
CITATION: SHREFFLER-GRANT ET AL. (2021) 14
CITATION: SISLER ET AL. (2019) 15
JBI CRITICAL APPRAISAL CHECKLIST FOR SYSTEMATIC REVIEWS AND RESEARCH SYNTHESES – TALLON ET AL. (2017) 16

Citation: Amiri and Zhao [1]

Category of study design	Methodological quality criteria	Responses			
		**Yes**	**No**	**Can't tell**	**Comments**
Screening questions (for all types)	S1. Are there clear research questions?	X			
	S2. Do the collected data allow to address the research questions?	X			
	Further appraisal may not be feasible or appropriate when the answer is 'No' or 'Can't tell' to one or both screening questions.				
1. Qualitative	1.1. Is the qualitative approach appropriate to answer the research question?	X			
	1.2. Are the qualitative data collection methods adequate to address the research question?	X			
	1.3. Are the findings adequately derived from the data?	X			
	1.4. Is the interpretation of results sufficiently substantiated by data?	X			
	1.5. Is there coherence between qualitative data sources, collection, analysis and interpretation?	X			
2. Quantitative randomized controlled trials	2.1. Is randomization appropriately performed?				
	2.2. Are the groups comparable at baseline?				
	2.3. Are there complete outcome data?				
	2.4. Are outcome assessors blinded to the intervention provided?				
	2.5 Did the participants adhere to the assigned intervention?				
3. Quantitative non-randomized	3.1. Are the participants representative of the target population?				
	3.2. Are measurements appropriate regarding both the outcome and intervention (or exposure)?				
	3.3. Are there complete outcome data?				
	3.4. Are the confounders accounted for in the design and analysis?				
	3.5. During the study period, is the intervention administered (or exposure occurred) as intended				
4. Quantitative descriptive	4.1. Is the sampling strategy relevant to address the research question?			X	
	4.2. Is the sample representative of the target population?	X			
	4.3. Are the measurements appropriate?	X			
	4.4. Is the risk of nonresponse bias low?			X	
	4.5. Is the statistical analysis appropriate to answer the research question?	X			
5. Mixed methods	5.1. Is there an adequate rationale for using a mixed methods design to address the research question?	X			
	5.2. Are the different components of the study effectively integrated to answer the research question?	X			
	5.3. Are the outputs of the integration of qualitative and quantitative components adequately interpreted?	X			
	5.4. Are divergences and inconsistencies between quantitative and qualitative results adequately addressed?	X			
	5.5. Do the different components of the study adhere to the quality criteria of each tradition of the methods involved?	X			

Citation: Barboza et al. [2]

**Category of study design**	**Methodological quality criteria**	**Responses**			
		**Yes**	**No**	**Can't tell**	**Comments**
Screening questions (for all types)	S1. Are there clear research questions?	X			
	S2. Do the collected data allow to address the research questions?	X			
	Further appraisal may not be feasible or appropriate when the answer is 'No' or 'Can't tell' to one or both screening questions.				
1. Qualitative	1.1. Is the qualitative approach appropriate to answer the research question?	X			
	1.2. Are the qualitative data collection methods adequate to address the research question?	X			
	1.3. Are the findings adequately derived from the data?	X			
	1.4. Is the interpretation of results sufficiently substantiated by data?	X			
	1.5. Is there coherence between qualitative data sources, collection, analysis and interpretation?	X			
2. Quantitative randomized controlled trials	2.1. Is randomization appropriately performed?				
	2.2. Are the groups comparable at baseline?				
	2.3. Are there complete outcome data?				
	2.4. Are outcome assessors blinded to the intervention provided?				
	2.5 Did the participants adhere to the assigned intervention?				
3. Quantitative non-randomized	3.1. Are the participants representative of the target population?				
	3.2. Are measurements appropriate regarding both the outcome and intervention (or exposure)?				
	3.3. Are there complete outcome data?				
	3.4. Are the confounders accounted for in the design and analysis?				
	3.5. During the study period, is the intervention administered (or exposure occurred) as intended				
4. Quantitative descriptive	4.1. Is the sampling strategy relevant to address the research question?				
	4.2. Is the sample representative of the target population?				
	4.3. Are the measurements appropriate?				
	4.4. Is the risk of nonresponse bias low?				
	4.5. Is the statistical analysis appropriate to answer the research question?				
5. Mixed methods	5.1. Is there an adequate rationale for using a mixed methods design to address the research question?				
	5.2. Are the different components of the study effectively integrated to answer the research question?				
	5.3. Are the outputs of the integration of qualitative and quantitative components adequately interpreted?				
	5.4. Are divergences and inconsistencies between quantitative and qualitative results adequately addressed?				
	5.5. Do the different components of the study adhere to the quality criteria of each tradition of the methods involved?				

Citation: Brooks et al. [3]

Category of study design	Methodological quality criteria	Responses			
		Yes	No	Can't tell	Comments
Screening questions (for all types)	S1. Are there clear research questions?	X			
	S2. Do the collected data allow to address the research questions?	X			
	Further appraisal may not be feasible or appropriate when the answer is 'No' or 'Can't tell' to one or both screening questions.				
1. Qualitative	1.1. Is the qualitative approach appropriate to answer the research question?	X			
	1.2. Are the qualitative data collection methods adequate to address the research question?	X			
	1.3. Are the findings adequately derived from the data?	X			
	1.4. Is the interpretation of results sufficiently substantiated by data?	X			
	1.5. Is there coherence between qualitative data sources, collection, analysis and interpretation?	X			
2. Quantitative randomized controlled trials	2.1. Is randomization appropriately performed?				
	2.2. Are the groups comparable at baseline?				
	2.3. Are there complete outcome data?				
	2.4. Are outcome assessors blinded to the intervention provided?				
	2.5 Did the participants adhere to the assigned intervention?				
3. Quantitative non-randomized	3.1. Are the participants representative of the target population?				
	3.2. Are measurements appropriate regarding both the outcome and intervention (or exposure)?				
	3.3. Are there complete outcome data?				
	3.4. Are the confounders accounted for in the design and analysis?				
	3.5. During the study period, is the intervention administered (or exposure occurred) as intended				
4. Quantitative descriptive	4.1. Is the sampling strategy relevant to address the research question?				
	4.2. Is the sample representative of the target population?				
	4.3. Are the measurements appropriate?				
	4.4. Is the risk of nonresponse bias low?				
	4.5. Is the statistical analysis appropriate to answer the research question?				
5. Mixed methods	5.1. Is there an adequate rationale for using a mixed methods design to address the research question?				
	5.2. Are the different components of the study effectively integrated to answer the research question?				
	5.3. Are the outputs of the integration of qualitative and quantitative components adequately interpreted?				
	5.4. Are divergences and inconsistencies between quantitative and qualitative results adequately addressed?				
	5.5. Do the different components of the study adhere to the quality criteria of each tradition of the methods involved?				

Citation: Browne-Yung et al. [4]

Category of study design	Methodological quality criteria	Responses			
		Yes	No	Can't tell	Comments
Screening questions (for all types)	S1. Are there clear research questions?	X			
	S2. Do the collected data allow to address the research questions?	X			
	Further appraisal may not be feasible or appropriate when the answer is 'No' or 'Can't tell' to one or both screening questions.				
1. Qualitative	1.1. Is the qualitative approach appropriate to answer the research question?	X			
	1.2. Are the qualitative data collection methods adequate to address the research question?	X			
	1.3. Are the findings adequately derived from the data?	X			
	1.4. Is the interpretation of results sufficiently substantiated by data?	X			
	1.5. Is there coherence between qualitative data sources, collection, analysis and interpretation?	X			
2. Quantitative randomized controlled trials	2.1. Is randomization appropriately performed?				
	2.2. Are the groups comparable at baseline?				
	2.3. Are there complete outcome data?				
	2.4. Are outcome assessors blinded to the intervention provided?				
	2.5 Did the participants adhere to the assigned intervention?				
3. Quantitative non-randomized	3.1. Are the participants representative of the target population?				
	3.2. Are measurements appropriate regarding both the outcome and intervention (or exposure)?				
	3.3. Are there complete outcome data?				
	3.4. Are the confounders accounted for in the design and analysis?				
	3.5. During the study period, is the intervention administered (or exposure occurred) as intended				
4. Quantitative descriptive	4.1. Is the sampling strategy relevant to address the research question?				
	4.2. Is the sample representative of the target population?				
	4.3. Are the measurements appropriate?				
	4.4. Is the risk of nonresponse bias low?				
	4.5. Is the statistical analysis appropriate to answer the research question?				
5. Mixed methods	5.1. Is there an adequate rationale for using a mixed methods design to address the research question?				
	5.2. Are the different components of the study effectively integrated to answer the research question?				
	5.3. Are the outputs of the integration of qualitative and quantitative components adequately interpreted?				
	5.4. Are divergences and inconsistencies between quantitative and qualitative results adequately addressed?				
	5.5. Do the different components of the study adhere to the quality criteria of each tradition of the methods involved?				

Citation: Dodge et al. [5]

**Category of study design**	**Methodological quality criteria**	**Responses**			
		**Yes**	**No**	**Can't tell**	**Comments**
Screening questions (for all types)	S1. Are there clear research questions?	X			
	S2. Do the collected data allow to address the research questions?	X			
	Further appraisal may not be feasible or appropriate when the answer is 'No' or 'Can't tell' to one or both screening questions.				
1. Qualitative	1.1. Is the qualitative approach appropriate to answer the research question?				
	1.2. Are the qualitative data collection methods adequate to address the research question?				
	1.3. Are the findings adequately derived from the data?				
	1.4. Is the interpretation of results sufficiently substantiated by data?				
	1.5. Is there coherence between qualitative data sources, collection, analysis and interpretation?				
2. Quantitative randomized controlled trials	2.1. Is randomization appropriately performed?	X			
	2.2. Are the groups comparable at baseline?	X			
	2.3. Are there complete outcome data?	X			
	2.4. Are outcome assessors blinded to the intervention provided?	X			
	2.5 Did the participants adhere to the assigned intervention?	X			
3. Quantitative non-randomized	3.1. Are the participants representative of the target population?				
	3.2. Are measurements appropriate regarding both the outcome and intervention (or exposure)?				
	3.3. Are there complete outcome data?				
	3.4. Are the confounders accounted for in the design and analysis?				
	3.5. During the study period, is the intervention administered (or exposure occurred) as intended				
4. Quantitative descriptive	4.1. Is the sampling strategy relevant to address the research question?				
	4.2. Is the sample representative of the target population?				
	4.3. Are the measurements appropriate?				
	4.4. Is the risk of nonresponse bias low?				
	4.5. Is the statistical analysis appropriate to answer the research question?				
5. Mixed methods	5.1. Is there an adequate rationale for using a mixed methods design to address the research question?				
	5.2. Are the different components of the study effectively integrated to answer the research question?				
	5.3. Are the outputs of the integration of qualitative and quantitative components adequately interpreted?				
	5.4. Are divergences and inconsistencies between quantitative and qualitative results adequately addressed?				
	5.5. Do the different components of the study adhere to the quality criteria of each tradition of the methods involved?				

Citation: Galletly et al. [6]

Category of study design	Methodological quality criteria	Responses			
		Yes	No	Can't tell	Comments
Screening questions (for all types)	S1. Are there clear research questions?	X			
	S2. Do the collected data allow to address the research questions?	X			
	Further appraisal may not be feasible or appropriate when the answer is 'No' or 'Can't tell' to one or both screening questions.				
1. Qualitative	1.1. Is the qualitative approach appropriate to answer the research question?				
	1.2. Are the qualitative data collection methods adequate to address the research question?				
	1.3. Are the findings adequately derived from the data?				
	1.4. Is the interpretation of results sufficiently substantiated by data?				
	1.5. Is there coherence between qualitative data sources, collection, analysis and interpretation?				
2. Quantitative randomized controlled trials	2.1. Is randomization appropriately performed?				
	2.2. Are the groups comparable at baseline?				
	2.3. Are there complete outcome data?				
	2.4. Are outcome assessors blinded to the intervention provided?				
	2.5 Did the participants adhere to the assigned intervention?				
3. Quantitative non-randomized	3.1. Are the participants representative of the target population?				
	3.2. Are measurements appropriate regarding both the outcome and intervention (or exposure)?				
	3.3. Are there complete outcome data?				
	3.4. Are the confounders accounted for in the design and analysis?				
	3.5. During the study period, is the intervention administered (or exposure occurred) as intended				
4. Quantitative descriptive	4.1. Is the sampling strategy relevant to address the research question?	X			
	4.2. Is the sample representative of the target population?			X	
	4.3. Are the measurements appropriate?	X			
	4.4. Is the risk of nonresponse bias low?			X	
	4.5. Is the statistical analysis appropriate to answer the research question?	X			
5. Mixed methods	5.1. Is there an adequate rationale for using a mixed methods design to address the research question?				
	5.2. Are the different components of the study effectively integrated to answer the research question?				
	5.3. Are the outputs of the integration of qualitative and quantitative components adequately interpreted?				
	5.4. Are divergences and inconsistencies between quantitative and qualitative results adequately addressed?				
	5.5. Do the different components of the study adhere to the quality criteria of each tradition of the methods involved?				

Citation: Godecker et al. [7]

Category of study design	Methodological quality criteria	Responses			
		Yes	No	Can't tell	Comments
Screening questions (for all types)	S1. Are there clear research questions?	X			
	S2. Do the collected data allow to address the research questions?	X			
	Further appraisal may not be feasible or appropriate when the answer is 'No' or 'Can't tell' to one or both screening questions.				
1. Qualitative	1.1. Is the qualitative approach appropriate to answer the research question?				
	1.2. Are the qualitative data collection methods adequate to address the research question?				
	1.3. Are the findings adequately derived from the data?				
	1.4. Is the interpretation of results sufficiently substantiated by data?				
	1.5. Is there coherence between qualitative data sources, collection, analysis and interpretation?				
2. Quantitative randomized controlled trials	2.1. Is randomization appropriately performed?				
	2.2. Are the groups comparable at baseline?				
	2.3. Are there complete outcome data?				
	2.4. Are outcome assessors blinded to the intervention provided?				
	2.5 Did the participants adhere to the assigned intervention?				
3. Quantitative non-randomized	3.1. Are the participants representative of the target population?	X			
	3.2. Are measurements appropriate regarding both the outcome and intervention (or exposure)?	X			
	3.3. Are there complete outcome data?	X			
	3.4. Are the confounders accounted for in the design and analysis?	X			
	3.5. During the study period, is the intervention administered (or exposure occurred) as intended	X			
4. Quantitative descriptive	4.1. Is the sampling strategy relevant to address the research question?				
	4.2. Is the sample representative of the target population?				
	4.3. Are the measurements appropriate?				
	4.4. Is the risk of nonresponse bias low?				
	4.5. Is the statistical analysis appropriate to answer the research question?				
5. Mixed methods	5.1. Is there an adequate rationale for using a mixed methods design to address the research question?				
	5.2. Are the different components of the study effectively integrated to answer the research question?				
	5.3. Are the outputs of the integration of qualitative and quantitative components adequately interpreted?				
	5.4. Are divergences and inconsistencies between quantitative and qualitative results adequately addressed?				
	5.5. Do the different components of the study adhere to the quality criteria of each tradition of the methods involved?				

Citation: Gruβ et al. [8]

**Category of study design**	**Methodological quality criteria**	**Responses**			
		**Yes**	**No**	**Can't tell**	**Comments**
Screening questions (for all types)	S1. Are there clear research questions?	X			
	S2. Do the collected data allow to address the research questions?	X			
	Further appraisal may not be feasible or appropriate when the answer is 'No' or 'Can't tell' to one or both screening questions.				
1. Qualitative	1.1. Is the qualitative approach appropriate to answer the research question?	X			
	1.2. Are the qualitative data collection methods adequate to address the research question?	X			
	1.3. Are the findings adequately derived from the data?	X			
	1.4. Is the interpretation of results sufficiently substantiated by data?	X			
	1.5. Is there coherence between qualitative data sources, collection, analysis and interpretation?	X			
2. Quantitative randomized controlled trials	2.1. Is randomization appropriately performed?				
	2.2. Are the groups comparable at baseline?				
	2.3. Are there complete outcome data?				
	2.4. Are outcome assessors blinded to the intervention provided?				
	2.5 Did the participants adhere to the assigned intervention?				
3. Quantitative non-randomized	3.1. Are the participants representative of the target population?				
	3.2. Are measurements appropriate regarding both the outcome and intervention (or exposure)?				
	3.3. Are there complete outcome data?				
	3.4. Are the confounders accounted for in the design and analysis?				
	3.5. During the study period, is the intervention administered (or exposure occurred) as intended				
4. Quantitative descriptive	4.1. Is the sampling strategy relevant to address the research question?				
	4.2. Is the sample representative of the target population?				
	4.3. Are the measurements appropriate?				
	4.4. Is the risk of nonresponse bias low?				
	4.5. Is the statistical analysis appropriate to answer the research question?				
5. Mixed methods	5.1. Is there an adequate rationale for using a mixed methods design to address the research question?				
	5.2. Are the different components of the study effectively integrated to answer the research question?				
	5.3. Are the outputs of the integration of qualitative and quantitative components adequately interpreted?				
	5.4. Are divergences and inconsistencies between quantitative and qualitative results adequately addressed?				
	5.5. Do the different components of the study adhere to the quality criteria of each tradition of the methods involved?				

Citation: Hornor et al. [9]

Category of study design	Methodological quality criteria	Responses			
		Yes	No	Can't tell	Comments
Screening questions (for all types)	S1. Are there clear research questions?	X			
	S2. Do the collected data allow to address the research questions?	X			
	Further appraisal may not be feasible or appropriate when the answer is 'No' or 'Can't tell' to one or both screening questions.				
1. Qualitative	1.1. Is the qualitative approach appropriate to answer the research question?				
	1.2. Are the qualitative data collection methods adequate to address the research question?				
	1.3. Are the findings adequately derived from the data?				
	1.4. Is the interpretation of results sufficiently substantiated by data?				
	1.5. Is there coherence between qualitative data sources, collection, analysis and interpretation?				
2. Quantitative randomized controlled trials	2.1. Is randomization appropriately performed?				
	2.2. Are the groups comparable at baseline?				
	2.3. Are there complete outcome data?				
	2.4. Are outcome assessors blinded to the intervention provided?				
	2.5 Did the participants adhere to the assigned intervention?				
3. Quantitative non-randomized	3.1. Are the participants representative of the target population?				
	3.2. Are measurements appropriate regarding both the outcome and intervention (or exposure)?				
	3.3. Are there complete outcome data?				
	3.4. Are the confounders accounted for in the design and analysis?				
	3.5. During the study period, is the intervention administered (or exposure occurred) as intended				
4. Quantitative descriptive	4.1. Is the sampling strategy relevant to address the research question?	X			
	4.2. Is the sample representative of the target population?		X		
	4.3. Are the measurements appropriate?	X			
	4.4. Is the risk of nonresponse bias low?		X		
	4.5. Is the statistical analysis appropriate to answer the research question?	X			
5. Mixed methods	5.1. Is there an adequate rationale for using a mixed methods design to address the research question?				
	5.2. Are the different components of the study effectively integrated to answer the research question?				
	5.3. Are the outputs of the integration of qualitative and quantitative components adequately interpreted?				
	5.4. Are divergences and inconsistencies between quantitative and qualitative results adequately addressed?				
	5.5. Do the different components of the study adhere to the quality criteria of each tradition of the methods involved?				

Citation: McCune et al. [10]

Category of study design	Methodological quality criteria	Responses			
		**Yes**	**No**	**Can't tell**	**Comments**
Screening questions (for all types)	S1. Are there clear research questions?	X			
	S2. Do the collected data allow to address the research questions?	X			
	Further appraisal may not be feasible or appropriate when the answer is 'No' or 'Can't tell' to one or both screening questions.				
1. Qualitative	1.1. Is the qualitative approach appropriate to answer the research question?				
	1.2. Are the qualitative data collection methods adequate to address the research question?				
	1.3. Are the findings adequately derived from the data?				
	1.4. Is the interpretation of results sufficiently substantiated by data?				
	1.5. Is there coherence between qualitative data sources, collection, analysis and interpretation?				
2. Quantitative randomized controlled trials	2.1. Is randomization appropriately performed?				
	2.2. Are the groups comparable at baseline?				
	2.3. Are there complete outcome data?				
	2.4. Are outcome assessors blinded to the intervention provided?				
	2.5 Did the participants adhere to the assigned intervention?				
3. Quantitative non-randomized	3.1. Are the participants representative of the target population?				
	3.2. Are measurements appropriate regarding both the outcome and intervention (or exposure)?				
	3.3. Are there complete outcome data?				
	3.4. Are the confounders accounted for in the design and analysis?				
	3.5. During the study period, is the intervention administered (or exposure occurred) as intended				
4. Quantitative descriptive	4.1. Is the sampling strategy relevant to address the research question?	X			
	4.2. Is the sample representative of the target population?	X			
	4.3. Are the measurements appropriate?	X			
	4.4. Is the risk of nonresponse bias low?	X			
	4.5. Is the statistical analysis appropriate to answer the research question?	X			
5. Mixed methods	5.1. Is there an adequate rationale for using a mixed methods design to address the research question?				
	5.2. Are the different components of the study effectively integrated to answer the research question?				
	5.3. Are the outputs of the integration of qualitative and quantitative components adequately interpreted?				
	5.4. Are divergences and inconsistencies between quantitative and qualitative results adequately addressed?				
	5.5. Do the different components of the study adhere to the quality criteria of each tradition of the methods involved?				

Citation: Monsen et al. [11]

Category of study design	Methodological quality criteria	Responses			
		Yes	No	Can't tell	Comments
Screening questions (for all types)	S1. Are there clear research questions?	X			
	S2. Do the collected data allow to address the research questions?	X			
	Further appraisal may not be feasible or appropriate when the answer is 'No' or 'Can't tell' to one or both screening questions.				
1. Qualitative	1.1. Is the qualitative approach appropriate to answer the research question?				
	1.2. Are the qualitative data collection methods adequate to address the research question?				
	1.3. Are the findings adequately derived from the data?				
	1.4. Is the interpretation of results sufficiently substantiated by data?				
	1.5. Is there coherence between qualitative data sources, collection, analysis and interpretation?				
2. Quantitative randomized controlled trials	2.1. Is randomization appropriately performed?				
	2.2. Are the groups comparable at baseline?				
	2.3. Are there complete outcome data?				
	2.4. Are outcome assessors blinded to the intervention provided?				
	2.5 Did the participants adhere to the assigned intervention?				
3. Quantitative non-randomized	3.1. Are the participants representative of the target population?				
	3.2. Are measurements appropriate regarding both the outcome and intervention (or exposure)?				
	3.3. Are there complete outcome data?				
	3.4. Are the confounders accounted for in the design and analysis?				
	3.5. During the study period, is the intervention administered (or exposure occurred) as intended				
4. Quantitative descriptive	4.1. Is the sampling strategy relevant to address the research question?	X			
	4.2. Is the sample representative of the target population?	X			
	4.3. Are the measurements appropriate?	X			
	4.4. Is the risk of nonresponse bias low?	X			
	4.5. Is the statistical analysis appropriate to answer the research question?	X			
5. Mixed methods	5.1. Is there an adequate rationale for using a mixed methods design to address the research question?				
	5.2. Are the different components of the study effectively integrated to answer the research question?				
	5.3. Are the outputs of the integration of qualitative and quantitative components adequately interpreted?				
	5.4. Are divergences and inconsistencies between quantitative and qualitative results adequately addressed?				
	5.5. Do the different components of the study adhere to the quality criteria of each tradition of the methods involved?				

Citation: Purkey et al. [12]

Category of study design	Methodological quality criteria	Responses			
		Yes	No	Can't tell	Comments
Screening questions (for all types)	S1. Are there clear research questions?	X			
	S2. Do the collected data allow to address the research questions?	X			
	Further appraisal may not be feasible or appropriate when the answer is 'No' or 'Can't tell' to one or both screening questions.				
1. Qualitative	1.1. Is the qualitative approach appropriate to answer the research question?	X			
	1.2. Are the qualitative data collection methods adequate to address the research question?	X			
	1.3. Are the findings adequately derived from the data?	X			
	1.4. Is the interpretation of results sufficiently substantiated by data?	X			
	1.5. Is there coherence between qualitative data sources, collection, analysis and interpretation?	X			
2. Quantitative randomized controlled trials	2.1. Is randomization appropriately performed?				
	2.2. Are the groups comparable at baseline?				
	2.3. Are there complete outcome data?				
	2.4. Are outcome assessors blinded to the intervention provided?				
	2.5 Did the participants adhere to the assigned intervention?				
3. Quantitative non-randomized	3.1. Are the participants representative of the target population?				
	3.2. Are measurements appropriate regarding both the outcome and intervention (or exposure)?				
	3.3. Are there complete outcome data?				
	3.4. Are the confounders accounted for in the design and analysis?				
	3.5. During the study period, is the intervention administered (or exposure occurred) as intended				
4. Quantitative descriptive	4.1. Is the sampling strategy relevant to address the research question?	X			
	4.2. Is the sample representative of the target population?			X	
	4.3. Are the measurements appropriate?	X			
	4.4. Is the risk of nonresponse bias low?			X	
	4.5. Is the statistical analysis appropriate to answer the research question?	X			
5. Mixed methods	5.1. Is there an adequate rationale for using a mixed methods design to address the research question?	X			
	5.2. Are the different components of the study effectively integrated to answer the research question?	X			
	5.3. Are the outputs of the integration of qualitative and quantitative components adequately interpreted?	X			
	5.4. Are divergences and inconsistencies between quantitative and qualitative results adequately addressed?	X			
	5.5. Do the different components of the study adhere to the quality criteria of each tradition of the methods involved?	X			

Citation: Shreffler-Grant et al. [13]

Category of study design	Methodological quality criteria	Responses			
		**Yes**	**No**	**Can't tell**	**Comments**
Screening questions (for all types)	S1. Are there clear research questions?	X			
	S2. Do the collected data allow to address the research questions?	X			
	Further appraisal may not be feasible or appropriate when the answer is 'No' or 'Can't tell' to one or both screening questions.				
1. Qualitative	1.1. Is the qualitative approach appropriate to answer the research question?				
	1.2. Are the qualitative data collection methods adequate to address the research question?				
	1.3. Are the findings adequately derived from the data?				
	1.4. Is the interpretation of results sufficiently substantiated by data?				
	1.5. Is there coherence between qualitative data sources, collection, analysis and interpretation?				
2. Quantitative randomized controlled trials	2.1. Is randomization appropriately performed?				
	2.2. Are the groups comparable at baseline?				
	2.3. Are there complete outcome data?				
	2.4. Are outcome assessors blinded to the intervention provided?				
	2.5 Did the participants adhere to the assigned intervention?				
3. Quantitative non-randomized	3.1. Are the participants representative of the target population?			X	
	3.2. Are measurements appropriate regarding both the outcome and intervention (or exposure)?	X			
	3.3. Are there complete outcome data?	X			
	3.4. Are the confounders accounted for in the design and analysis?			X	
	3.5. During the study period, is the intervention administered (or exposure occurred) as intended	X			
4. Quantitative descriptive	4.1. Is the sampling strategy relevant to address the research question?				
	4.2. Is the sample representative of the target population?				
	4.3. Are the measurements appropriate?				
	4.4. Is the risk of nonresponse bias low?				
	4.5. Is the statistical analysis appropriate to answer the research question?				
5. Mixed methods	5.1. Is there an adequate rationale for using a mixed methods design to address the research question?				
	5.2. Are the different components of the study effectively integrated to answer the research question?				
	5.3. Are the outputs of the integration of qualitative and quantitative components adequately interpreted?				
	5.4. Are divergences and inconsistencies between quantitative and qualitative results adequately addressed?				
	5.5. Do the different components of the study adhere to the quality criteria of each tradition of the methods involved?				

Citation: Sisler et al. [14]

Category of study design	Methodological quality criteria	Responses			
		Yes	No	Can't tell	Comments
Screening questions (for all types)	S1. Are there clear research questions?	X			
	S2. Do the collected data allow to address the research questions?	X			
	Further appraisal may not be feasible or appropriate when the answer is 'No' or 'Can't tell' to one or both screening questions.				
1. Qualitative	1.1. Is the qualitative approach appropriate to answer the research question?	X			
	1.2. Are the qualitative data collection methods adequate to address the research question?	X			
	1.3. Are the findings adequately derived from the data?	X			
	1.4. Is the interpretation of results sufficiently substantiated by data?	X			
	1.5. Is there coherence between qualitative data sources, collection, analysis and interpretation?	X			
2. Quantitative randomized controlled trials	2.1. Is randomization appropriately performed?				
	2.2. Are the groups comparable at baseline?				
	2.3. Are there complete outcome data?				
	2.4. Are outcome assessors blinded to the intervention provided?				
	2.5 Did the participants adhere to the assigned intervention?				
3. Quantitative non-randomized	3.1. Are the participants representative of the target population?				
	3.2. Are measurements appropriate regarding both the outcome and intervention (or exposure)?				
	3.3. Are there complete outcome data?				
	3.4. Are the confounders accounted for in the design and analysis?				
	3.5. During the study period, is the intervention administered (or exposure occurred) as intended				
4. Quantitative descriptive	4.1. Is the sampling strategy relevant to address the research question?				
	4.2. Is the sample representative of the target population?				
	4.3. Are the measurements appropriate?				
	4.4. Is the risk of nonresponse bias low?				
	4.5. Is the statistical analysis appropriate to answer the research question?				
5. Mixed methods	5.1. Is there an adequate rationale for using a mixed methods design to address the research question?				
	5.2. Are the different components of the study effectively integrated to answer the research question?				
	5.3. Are the outputs of the integration of qualitative and quantitative components adequately interpreted?				
	5.4. Are divergences and inconsistencies between quantitative and qualitative results adequately addressed?				
	5.5. Do the different components of the study adhere to the quality criteria of each tradition of the methods involved?				

JBI Critical Appraisal Checklist for systematic reviews and research syntheses – Tallon et al. [15]

Reviewer: Deirdre McGowan (DM); Christine Stirling (CS). Date: DM 1/3/21; CS 15/3/21

Author: Tallon et al. Year: 2017 Record Number: 14

	Yes	No	Unclear	Not applicable
1. Is the review question clearly and explicitly stated?	X	□	□	□
2. Were the inclusion criteria appropriate for the review question?	X	□	□	□
3. Was the search strategy appropriate?	X	□	□	□
4. Were the sources and resources used to search for studies adequate?	X	□	□	□
5. Were the criteria for appraising studies appropriate?	X	□	□	□
6. Was critical appraisal conducted by two or more reviewers independently?	X	□	□	□
7. Were there methods to minimize errors in data extraction?	X	□	□	□
8. Were the methods used to combine studies appropriate?	X	□	□	□
9. Was the likelihood of publication bias assessed?	□	□	X	□
10. Were recommendations for policy and/or practice supported by the reported data?	X	□	□	□
11. Were the specific directives for new research appropriate?	X	□	□	□

Overall appraisal: Include X Exclude □ Seek further info □

Comments (Including reason for exclusion)

© JBI, 2020. All rights reserved. JBI grants use of these tools for research purposes only. All other enquiries should be sent to jbisynthesis@adelaide.edu.

1. Amiri A, Zhao S. Working with an environmental justice community: Nurse observation, assessment, and intervention. Nursing forum. 2019;54(2):270-9.
2. Barboza M, Kulane A, Burstrom B, Marttila A. A better start for health equity? Qualitative content analysis of implementation of extended postnatal home visiting in a disadvantaged area in Sweden. International Journal for Equity in Health. 2018;17(1).
3. Brooks C, Ballinger C, Nutbeam D, Mander C, Adams J. Nursing and allied health professionals' views about using health literacy screening tools and a universal precautions approach to communication with older adults: a qualitative study. Disability & Rehabilitation. 2020;42(13):1819-25.
4. Browne-Yung K, Freeman T, Battersby MW, McEvoy DR, Baum F. Developing a screening tool to recognise social determinants of health in Australian clinical settings. Public Health Research and Practice. 2019;29(4):1-6.
5. Dodge KA, Goodman WB, Murphy RA, O'Donnell K, Sato J, Guptill S. Implementation and Randomized Controlled Trial Evaluation of Universal Postnatal Nurse Home Visiting. American Journal of Public Health. 2014;104(S1):S136-S43.
6. Galletly C, Neaves A, Burton C, Liu D, Denson LA. Evaluating health literacy in people with mental illness using the Test of Functional Health Literacy in Adults. Nursing Outlook. 2012;60(5):316-21.
7. Godecker AL, Harrison PA, Sidebottom AC. Nurse versus community health worker identification of psychosocial risks in pregnancy through a structured interview. Journal of Health Care for the Poor & Underserved. 2013;24(4):1574-85.
8. Gruβ I, Bunce A, Davis J, Dambrun K, Cottrell E, Gold R. Initiating and Implementing Social Determinants of Health Data Collection in Community Health Centers. Population health management. 2020;24(1):52-8.
9. Hornor G, Bretl D, Chapman E, Herendeen P, Mitchel N, Mulvaney B, et al. Child Maltreatment Screening and Anticipatory Guidance: A Description of Pediatric Nurse Practitioner Practice Behaviors. Journal of Pediatric Healthcare. 2017;31(6):e35-e44.
10. McCune RL, Lee H, Pohl JM. Assessing health literacy in safety net primary care practices. Applied Nursing Research. 2016;29:188-94.
11. Monsen KA, Rudenick JM, Kapinos N, Warmbold K, McMahon SK, Schorr EN. Documentation of social determinants in electronic health records with and

without standardized terminologies: A comparative study. Proceedings of Singapore Healthcare. 2019;28(1):39-47.

12. Purkey E, Bayoumi I, Coo H, Maier A, Pinto AD, Olomola B, et al. Exploratory study of "real world" implementation of a clinical poverty tool in diverse family medicine and pediatric care settings. International Journal for Equity in Health. 2019;18(1):1-9.

13. Shreffler-Grant J, Nichols EG, Weinert C. Community-based Skill Building Intervention to Enhance Health Literacy Among Older Rural Adults. Western Journal of Nursing Research. 2020;00(0):1-9.

14. Sisler SM, Schapiro NA, Stephan L, Mejia J, Wallace AS. Consider the root of the problem: Increasing trainee skills at assessing and addressing social determinants of health. Translational Behavioral Medicine. 2019;9(3):523-32.

15. Tallon MM, Kendall GE, Priddis L, Newall F, Young J. Barriers to addressing social determinants of health in pediatric nursing practice: an integrative review. Journal of pediatric nursing. 2017;37:51-6.
